# Exposure to chronic mild stress prevents kappa opioid-mediated reinstatement of cocaine and nicotine place preference

**DOI:** 10.3389/fphar.2013.00096

**Published:** 2013-08-06

**Authors:** Ream Al-Hasani, Jordan G. McCall, Michael R. Bruchas

**Affiliations:** ^1^Basic Research Division, Department of Anesthesiology, Washington University School of MedicineSt. Louis, MO, USA; ^2^Department of Anatomy and Neurobiology, Washington University School of MedicineSt. Louis,MO, USA; ^3^Division of Biological and Biomedical Sciences, Washington University School of MedicineSt. Louis, MO, USA; ^4^Washington University Pain Center, Washington University School of MedicineSt. Louis, MO, USA

**Keywords:** kappa opioid receptor, cocaine, nicotine, stress, conditioned place preference

## Abstract

Stress increases the risk of drug abuse, causes relapse to drug seeking, and potentiates the rewarding properties of both nicotine and cocaine. Understanding the mechanisms by which stress regulates the rewarding properties of drugs of abuse provides valuable insight into potential treatments for drug abuse. Prior reports have demonstrated that stress causes dynorphin release, activating kappa opioid receptors (KOR) in monoamine circuits resulting in both potentiation and reinstatement of cocaine and nicotine conditioned place preference. Here we report that kappa opioid-dependent reinstatement of cocaine and nicotine place preference is reduced when the mice are exposed to a randomized chronic mild stress (CMS) regime prior to training in a conditioned place preference-reinstatement paradigm. The CMS schedule involves seven different stressors (removal of nesting for 24 h, 5 min forced swim stress at 15°C, 8 h food and water deprivation, damp bedding overnight, white noise, cage tilt, and disrupted home cage lighting) rotated over a 3-week period. This response is KOR-selective, as CMS does not protect against cocaine or nicotine drug-primed reinstatement. This protection from reinstatement is also observed following sub-chronic social defeat stress, where each mouse is placed in an aggressor mouse home cage for a period of 20 min over 5 days. In contrast, a single acute stressor resulted in a potentiation of KOR-induced reinstatement, as previously reported. Prior studies have shown that stress alters sensitivity to opioids and prior stress can influence the pharmacodynamics of the opioid receptor system. Together, these findings suggest that exposure to different forms of stress may cause a dysregulation of kappa opioid circuitry and that changes resulting from mild stress can have protective and adaptive effects against drug relapse.

## INTRODUCTION

It is well-established that stress increases the risk of drug abuse, relapse to drug seeking, and potentiates the rewarding properties of cocaine (Covington and Miczek, 2001; McLaughlin et al., 2003; Redila and Chavkin, 2008; Bruchas et al., 2010). Previous studies have implicated a critical role for dynorphin/kappa opioid systems in the mediation of stress-induced behaviors including reinstatement of drug seeking in both place preference and self-administration animal models. Studies have shown that stress-induced reinstatement to alcohol, cocaine, and nicotine seeking is absent in kappa opioid receptor (KOR) KO and dynorphin KO mice as well as following pretreatment with KOR antagonists (Beardsley et al., 2005; Redila and Chavkin, 2008; Walker and Koob, 2008; Walker et al., 2011; Jackson et al., 2013). However, while numerous models of acute stress reinstate drug seeking through dynorphin/KOR activation, the effects of prior repeated stress exposure, and various magnitudes of stress exposure on KOR-mediated reinstatement has not been examined.

The dynorphin/KOR system is composed of prodynorphin peptides and KOR, a seven-transmembrane spanning G_i/o_ protein-coupled receptor (GPCR). In addition to the classical inhibitor effects on adenylate cyclase activity, KOR also couples to mitogen-activated protein kinase pathways (Bruchas et al., 2010; Al-Hasani and Bruchas, 2011) to mediate various behavioral effects (Bruchas et al., 2011; Potter et al., 2011). It is thought that stress causes dynorphin release activating KOR within monoamine nuclei (ventral tegmental area, dorsal raphe, locus coeruleus) and their projection targets (extended amygdala, nucleus accumbens, etc.; Wise, 2004; Zhang et al., 2005; Carlezon et al., 2006; Gehrke et al., 2008; Land et al., 2008, 2009; Ebner et al., 2010; Bruchas et al., 2011; Al-Hasani et al., 2013; Graziane et al., 2013). The KOR-mediated reduction in dopamine and serotonin activity results in dysphoria-like behavior that drives reinstatement of drug seeking to relieve this negative affective state. KORs are highly regulated via G-protein-coupled receptor kinase (GRK) phosphorylation and desensitization mechanisms. In opioid receptors this cellular regulatory system acts to remove receptor-G-protein activation, and promotes tolerance, arrestin signaling and/or receptor recycling (McLaughlin et al., 2004; Al-Hasani and Bruchas, 2011; Williams et al., 2013).

Chronic mild stress (CMS) is a widely adopted animal model for inducing depression- and anxiety-like behaviors because it mimics the unpredictable intermittent stress exposure that humans experience, for complete review see Hill et al. (2012). The model utilizes repeated, randomized stress events, over the course of several weeks to mimic the unpredictable nature of mild stress experience (Elizalde et al., 2008; Peng et al., 2012; Tye et al., 2013). Numerous studies have demonstrated that various types of CMS models act to alter neurotransmitter systems including monoamine (dopamine, serotonin, and norepinephrine), gamma-aminobutyric acid (GABA), and glutamatergic transmission (Rasheed et al., 2008; Vancassel et al., 2008; Tye et al., 2013). However, the role of CMS on opioid system regulation is not as well-known, with only a few reports showing that dynorphin and enkephalin opioid neuropeptide mRNA remain relatively unchanged in CMS models in various brain regions (Bertrand et al., 1997; Bergström et al., 2008). More recent reports have shown that repeated stress can dysregulate the effects of KOR signaling within dorsal raphe serotonergic circuits (Lemos et al., 2012a) but the functional consequences of repeated stress exposure or CMS compared to acute and sub-chronic stressors on kappa opioid-dependent behaviors has not been investigated.

Here we determined how different types of exposure to stress (acute, sub-chronic, and chronic) impact subsequent kappa opioid-mediated reinstatement of cocaine and nicotine place preference. We determined how different forms of stress, including a 3-week CMS, sub-chronic social defeat stress (SDS), and acute forced swim stress (FSS), impact kappa opioid-induced reinstatement. We found that the initial cocaine and nicotine conditioned place preference were unchanged in sub-chronic stress and CMS exposure, however, KOR-induced cocaine and nicotine reinstatement was absent in mice that were pre-exposed to CMS, in contrast to acute stress, which caused potentiated KOR-reinstatement. In addition, we found that cocaine and nicotine drug-primed reinstatement is not affected by pre-exposure to stress, suggesting that this protective ablation of KOR-induced reinstatement by CMS, selectively affects KOR-mediated behavioral responses.

## MATERIALS AND METHODS

### ANIMAL SUBJECTS

Male C57BL/6 wild-type mice, bred locally and maintained in Washington University mouse facility (20–30 g) were used for all experiments. All mice were group-housed within the Animal Core Facility at Washington University in St. Louis, given access to food pellets and water *ad libitum, *and maintained in specific pathogen-free housing. Mice were transferred at least 1 week before testing into a colony room adjacent to the behavioral testing room to acclimatize to the study environment and prevent stress during conditioning phases. Housing rooms were illuminated on a 12-h light/dark cycle with lights on at 7 AM. All animal procedures were approved by the Animal Care and Use Committee of Washington University in St. Louis, in accordance with the National Institutes of Health *Guide for the Care and Use of Laboratory Animals*.

### DRUGS

Cocaine HCl and racemic U50,488 methanesulfonate were provided by the National Institute on Drug Abuse and Drug Supply Program and in some instances Sigma Aldrich (St. Louis, MO, USA). Nicotine hydrogen tartrate salt was purchased from Sigma Aldrich (St. Louis, MO, USA), dissolved in phosphate buffered solution. The free base form of nicotine was used for calculating all injection doses. All drugs were dissolved in saline unless otherwise indicated.

### CONDITIONED PLACE PREFERENCE AND REINSTATEMENT PARADIGM

Mice were trained in an unbiased, balanced three-compartment conditioning apparatus and the reinstatement of cocaine place preference (CPP) paradigm was conducted as previously described (Land et al., 2009; Bruchas et al., 2011; Al-Hasani et al., 2013). On pre-conditioning day mice were allowed free access to all three chambers for 30 min (cocaine CPP) and 20 min (nicotine CPP). Time spent in each compartment was recorded with a video camera (ZR90; Canon) and analyzed using Ethovision 8.5 (Noldus). Mice were randomly assigned to saline and drug compartments and received a saline injection in the morning (10 ml/kg, s.c.) and cocaine (15 mg/kg, s.c.) or nicotine (0.5 mg/kg, s.c.; Jackson et al., 2013) injection in the afternoon, at least 4 h after the morning training on two consecutive days for nicotine conditioning and three consecutive days for cocaine conditioning. To test for cocaine place preference the mice were allowed free access to the three compartments. Scores were calculated by subtracting the time spent in the drug-paired compartment, post-test minus the pre-test. Mice were considered to have conditioned if the conditioning score was within 15–85% of total conditioning time, approximately 50 and 80% of mice reached this criteria for nicotine and cocaine conditioning, respectively. This was followed by 2 days (nicotine) or 3 days (cocaine) of extinction training during which saline (10 ml/kg, s.c.) was injected in both the morning and afternoon prior to placement into isolated conditioning compartments. Mice were then tested for extinction of place preference with free access to all three chambers. Mice were considered to have extinguished cocaine and nicotine preference if scores fell within 15% of their initial preference scores, approximately 98% of mice met this criteria. Mice that did not meet these criteria were excluded from the study and mice that met these criteria and extinguished continued on to the reinstatement phase.

On reinstatement test day mice were injected with KOR agonist, U50,488 (5 mg/kg, i.p.), placed in their home cage for 30 min (cocaine CPP) or 20 min (nicotine CPP) as previously described (Redila and Chavkin, 2008; Land et al., 2009; Al-Hasani et al., 2013), after which they were placed in the CPP apparatus and allowed free access to all three compartments for reinstatement expression. Reinstatement was measured as time (s) in drug-paired chamber on reinstatement test day minus time spent in drug-paired chamber following extinction training. On the following day, all mice were exposed to a priming injection of cocaine (15 mg/kg, s.c.) or nicotine (0.5 mg/kg, s.c.) to test for drug-primed reinstatement and placed in the apparatus with free access to all compartments. Reinstatement scores were calculated by subtracting the time spent in the cocaine or nicotine side post-reinstatement minus the extinction test as previously described (Land et al., 2009; Bruchas et al., 2011).

### ACUTE STRESS

During reinstatement (day 10) mice were subjected to FSS in which they were placed in an 18 cm deep bucket of water at 30°C and allowed to swim for upto 6 min. Mice were removed immediately if they appeared to be at risk of drowning. After a 5 min dry off and recovery period mice were injected with U50, 488 (5 mg/kg, i.p.) in the home cage for 30 min and then placed in the CPP chambers, with access to all three compartments to access reinstatement to cocaine place preference.

### SUB-CHRONIC SOCIAL DEFEAT STRESS

Social defeat stress was performed as previously described (Bruchas et al., 2011). From the afternoon of the conditioning post-test (day 5) until post-extinction day (day 9) mice were placed in the home cage of an aggressor mouse for a period of 20 min in the afternoon 2 h following extinction or post-testing (typically from 4 to 6 PM). Mice were monitored carefully for severe injury during SDS and were removed if necessary. No mice met this criterion in this study. In addition, as previously described (Land et al., 2009; Bruchas et al., 2011) all mice are observed and tracked to receive similar bouts of aggression and exhibit characteristic social defeat postures (McLaughlin et al., 2006; Land et al., 2009) to ensure similar stress exposure in each treatment group. On day 10 mice were injected with U50,488 (5 mg/kg, i.p.) in the home cage and then placed in the CPP chambers 30 min later to access reinstatement to cocaine seeking. On day 11, for drug-primed reinstatement, mice were injected with cocaine (15 mg/kg, i.p.) and immediately placed in the testing chambers.

### CHRONIC MILD STRESS PARADIGM

In order to model randomized mild stress exposure, we adapted a previously validated CMS paradigm. Stressors were randomly assigned during the 3-week stress period (outlined in **Table 1**) prior to conditioned place preference/reinstatement procedure. During reinstatement phase the mice were injected with U50,488 (5 mg/kg, i.p.) in the home cage (30 min) and then placed in the CPP chambers to assess reinstatement to cocaine place preference. On the following day, mice were injected with cocaine (15 mg/kg, s.c.) or nicotine (0.5 mg/kg, s.c.) priming injection and again allowed free access to all three chambers to determine drug prime-induced reinstatement of place preference.

**Table 1:** Three-week chronic mild stress schedule.

Week	Day 1	Day 2	Day 3	Day 4	Day 5	Day 6	Day 7
1	17:00 to next day: damp bedding	4 h white noise	8 h cage tilt	Continuous light 24 h	Removal of nesting 24 h	Food and water deprivation 8 h	Swimming at 4°C for 5 min
2	Continuous light 24 h	17:00 to next day: damp bedding	Food and water deprivation 8 h	Swimming at 4°C for 5 min	8 h cage tilt	4 h white noise	Removal of nesting 24 h
3	Swimming at 4°C for 5 min	Continuous light 24 h	8 h cage tilt	Removal of nesting 24 h	4 h white noise	Food and water deprivation 8 h	17:00 to next day: damp bedding

*Three weeks prior to cocaine or nicotine conditioned place preference-reinstatement paradigm the mice were randomly assigned the different stressors highlighted in the table. Paradigm is modified from Elizalde et al., 2008; Peng et al., 2012; and Tye etal. (2013).*

### LOCOMOTOR ACTIVITY

During the 3-day conditioning period and during reinstatement test days, locomotor activity was recorded as distance traveled (cm) throughout the 30 min period using video tracking (Canon) of animal movement and Ethovision 8.5 software analysis (Noldus). During the conditioning period the total distance (cm) on conditioning days are represented. During reinstatement post-test trials distance (cm) is represented as 5 min bins throughout the 30 min test period.

### DATA ANALYSES AND STATISTICS

Data were expressed as means ± SEM. All raw data were calculated via Ethovision video tracking and then place preference or locomotor data were calculated as described. Data were normally distributed, and differences between groups were determined using Student’s independent *t*-tests, one-way ANOVA, or two-way ANOVA as appropriate. ANOVA’s were followed by *post hoc* Bonferroni comparisons if the main effect was significant at *p* < 0.05. Statistical analyses were conducted using GraphPad Prism 5.0F (GraphPad, San Diego, CA, USA).

## RESULTS

### ACTIVATION OF KAPPA OPIOID RECEPTORS FOLLOWING ACUTE STRESS POTENTIATES REINSTATEMENT OF COCAINE PLACE PREFERENCE

**FIGURE 1:**
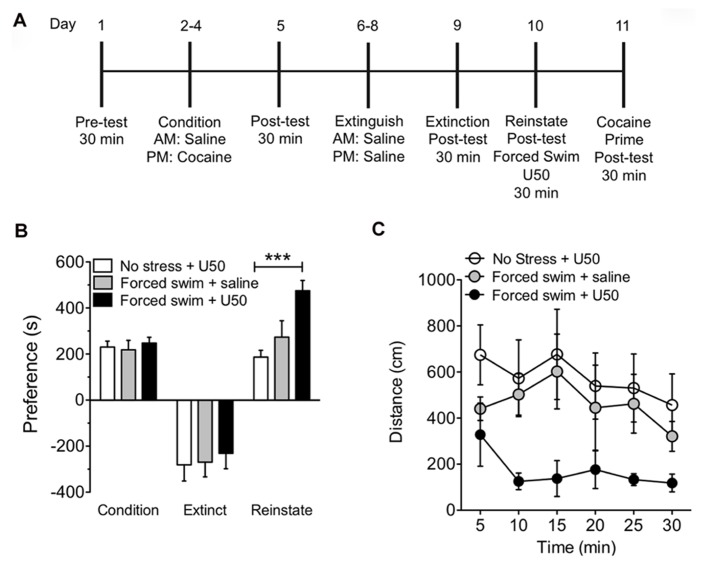
**Acute forced swim stress potentiates U50,488-induced reinstatement of cocaine place preference. (A)** Timeline of cocaine place preference-reinstatement experimental paradigm. **(B)** Cocaine preference scores (day 5), calculated as post-test minus pre-test on the cocaine-paired side (condition) and U50,488-induced (5 mg/kg, i.p.) reinstatement scores (day 10) of extinguished place preference (reinstate). Data show a significant potentiation in KOR-reinstatement following forced swim stress and U50,488. Groups are defined in the figures as their final reinstatement grouping (U50 = U50,488). Data represent the mean preference (s) ± SEM, *n* = 8–15 (***p < 0.001, no stress and U50 vs. forced swim stress and U50; one-way ANOVA followed by Bonferroni *post hoc* test). **(C)** Locomotor activity measured as distance traveled (cm) during the 30 min reinstatement trial. Data represent the mean preference (s) ± SEM, *n* = 8–15.

It has previously been shown that FSS is sufficient to induce reinstatement and potentiation to drug seeking in a KOR-dependent manner (McLaughlin et al., 2003; Schindler et al., 2012; Smith et al., 2012). Furthermore, stress-induced activation of KOR reinstates nicotine, alcohol, and cocaine seeking (Bruchas et al., 2010; Van Bockstaele et al., 2010; Wee and Koob, 2010; Graziane et al., 2013). In this study, we determined whether acute exposure to a single swim stress would prevent or potentiate a subsequent KOR-mediated reinstatement of CPP. Mice were subjected to cocaine conditioning, extinction, and reinstatement as described. On reinstatement day mice were exposed to swim stress for up to 6 min, were injected with KOR agonist U50,488 (5 mg/kg, i.p) 5 min following recovery, and then placed in the CPP chamber 30 min later (**Figure 1A**). Mice subjected to FSS prior to KOR activation by U50,488 showed significant and robust potentiation of reinstatement to cocaine seeking when compared to mice that were not subjected to FSS but that were injected with only U50,488 to induce reinstatement (**Figure 1B**, *n* = 8–15 (***p < 0.001, no stress and U50 vs. FSS and U50; one-way ANOVA, Bonferroni *post hoc* test). Locomotor activity during the reinstatement test was measured and the combination of FSS, followed by KOR-induced reinstatement to cocaine seeking showed a reduction in locomotor activity when compared to both the non-stressed group and the FSS alone group (**Figure 1C**). These data suggest that acute stress exposure induces a potentiation of KOR agonist-induced reinstatement of cocaine place preference.

### SUB-CHRONIC STRESS EXPOSURE BLOCKS SUBSEQUENT KOR AGONIST-INDUCED REINSTATEMENT OF COCAINE PLACE PREFERENCE

**FIGURE 2:**
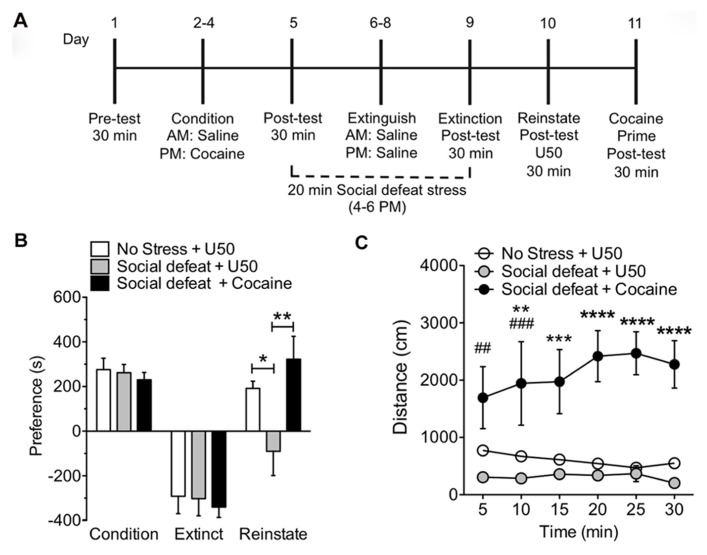
**Exposure to sub-chronic social defeat stress prevents U50,488-induced reinstatement of cocaine place preference**. **(A)** Timeline of cocaine place preference-reinstatement experiment. **(B)** Cocaine preference scores (day 5) calculated as post-test minus pre-test on the cocaine-paired side (condition) and U50,488-induced (5 mg/kg, i.p.) reinstatement scores (day 10) of extinguished place preference (reinstate), calculated as reinstatement test day minus extinction post-test day. Groups are defined in the figures as their final reinstatement grouping (U50 = U50,488). Data show a significant attenuation in KOR-reinstatement following sub-chronic social defeat stress. Data represent the mean preference (s) ± SEM, *n* = 12–16 (*p < 0.05, no stress and U50,488 vs. social defeat stress and U50,488, **p < 0.01, social defeat stress and U50,488 vs. social defeat stress and cocaine; one-way ANOVA followed by Bonferroni *post hoc* test). **(C)** Locomotor activity measured as distance traveled (cm) during the 30 min reinstatement trial. Data represent the mean preference (s) ± SEM, *n* = 12–16 (^##^*p* < 0.01 and ^###^*p* < 0.005, social defeat stress and U50,488 vs. social defeat stress and cocaine at 5 and 10 min time point, respectively; **p < 0.01, no stress and U50,488 vs. social defeat stress and U50,488 at 10 min time point; ***p < 0.005, social defeat stress and cocaine vs. both no stress and U50 and social defeat stress and U50,488 at 15 min; ****p < 0.001 social defeat stress and cocaine vs. both no stress and U50 and social defeat stress and U50,488 at 20, 25, and 30 min time points; two-way ANOVA followed by Bonferroni *post hoc* test).

To determine the effects of multiple rounds of stress exposure on KOR-induced reinstatement, mice were subjected to 20 min of SDS at the end of the day during extinction training and prior to the reinstatement phase. On day 10, the reinstatement test day, a single dose of U50,488 (5 mg/kg, i.p.) was administered to induce reinstatement of cocaine place preference (**Figure 2A**). Prior exposure to sub-chronic SDS resulted in a significant block of U50,488 induced reinstatement of cocaine place preference (**Figure 2B**: *p < 0.05, no stress and U50,488 vs. SDS and U50,488, **p < 0.01, SDS and U50,488 vs. SDS and cocaine; one-way ANOVA followed by Bonferroni *post hoc* test, *n* = 12–16). This inhibition of reinstatement was selective for activation of KOR because a priming injection of cocaine (15 mg/kg, s.c.) still caused significant reinstatement of cocaine CPP following exposure to the sub-chronic SDS paradigm (**Figure 2B**; **p < 0.01, SDS and U50 vs. SDS with cocaine, *n* = 12–16). We also measured locomotor activity during the 30 min reinstatement phase. Both the non-stress + U50,488 group and the SDS + U50,488 group show a similar KOR-mediated reduction in locomotor over the 30 min period, supporting the notion that prevention of reinstatement by pre-exposure to stress, is not due to additional alterations in mouse locomotor activity (**Figure 2C**). In contrast, as shown in previous studies (Reith, 1986; Tolliver et al., 1994; Giros et al., 1996; Sora et al., 1998; Zhang et al., 2002) locomotor activity was significantly elevated in mice reinstated with cocaine (^##^*p* < 0.01 and ^###^*p* < 0.005, SDS and U50,488 vs*.* SDS and cocaine at 5 and 10 min time point, respectively; **p < 0.01, no stress and U50,488 vs. SDS and U50,488 at 10 min time point; ***p < 0.005, SDS and cocaine vs. both no stress and U50 and SDS and U50,488 at 15 min; ****p < 0.001 SDS and cocaine vs. both no stress and U50 and SDS and U50,488 at 20, 25, and 30 min time points; two-way ANOVA followed by Bonferroni *post hoc* test, *n* = 12–16). Taken together, these data suggest that exposure to a sub-chronic repeated SDS causes a significant prevention of kappa agonist-induced reinstatement of cocaine place preference.

### CHRONIC MILD STRESS PROTECTS AGAINST U50,488-INDUCED REINSTATEMENT TO COCAINE AND NICOTINE PLACE PREFERENCE

**FIGURE 3:**
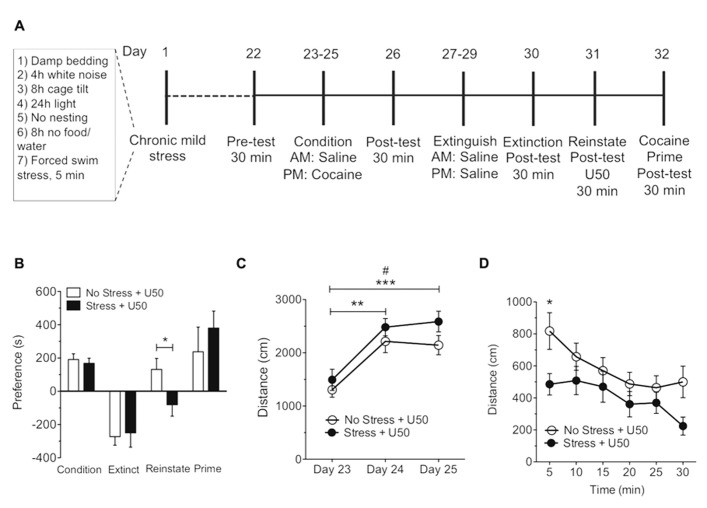
**xposure to chronic mild stress blocks U50,488-induced but not cocaine-primed reinstatement of cocaine place preference**. **(A)** Timeline of cocaine place preference-reinstatement experiment. **(B)** Cocaine preference scores (day 26), calculated as post-test minus pre-test on the cocaine-paired side (condition), U50,488-induced (5 mg/kg, i.p.) reinstatement scores (reinstate, day 31), or cocaine-induced (15 mg/kg, i.p) reinstatement (prime, day 32) of extinguished place preference. Data show a significant attenuation in U50,488-induced but not cocaine-primed reinstatement to cocaine seeking following chronic mild stress. Groups are defined in the figures as their final reinstatement grouping (U50 = U50,488).Data represent the mean preference (s) ± SEM, *n* = 7 (*p < 0.05, no stress and U50,488 vs. stress and U50,488; Unpaired Student’s *t*-test). **(C)** Locomotor activity measured as distance traveled (cm) during three cocaine conditioning days. Data represent the mean preference (s) ± SEM, *n* = 7 (^#^*p* < 0.05 day 23 vs. day 25 in the no stress group; **p < 0.01day 23 vs. day 24 and ***p < 0.005 day 23 vs. day 25 between stress group; one-way ANOVA followed by Bonferroni *post hoc* test). **(D)** Locomotor activity measured as distance traveled (cm) during reinstatement. Data represent the mean preference (s) ± SEM, *n* = 7 (*p < 0.05 at 5 min time point in the no stress group vs. CMS group; two-way ANOVA followed by Bonferroni *post hoc* test).

**FIGURE 4:**
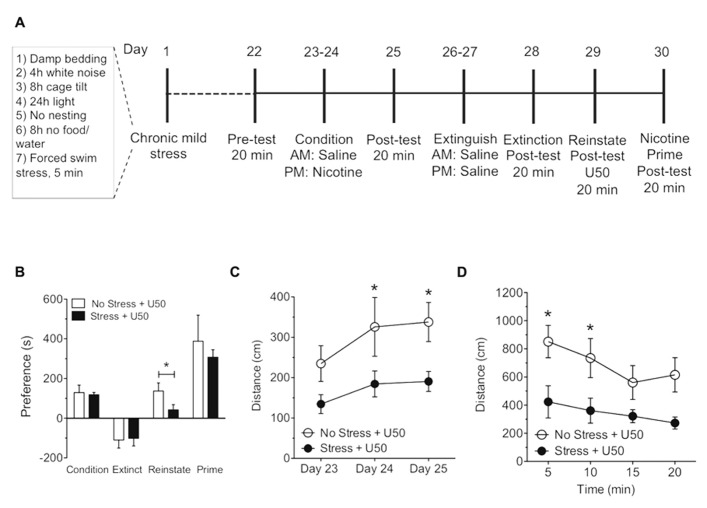
**Exposure to chronic mild stress blocks U50,488-induced but not nicotine-primed reinstatement of nicotine place preference**. **(A)** Timeline of nicotine place preference-reinstatement experiment. **(B)** Nicotine preference scores (day 26), calculated as post-test minus pre-test on the nicotine-paired side(condition), U50,488-induced (5 mg/kg, i.p.; reinstate, day 31) and nicotine-induced (0.5mg/kg, i.p) reinstatement (prime, day 32) scores of extinguished place preference. Data show a significant reinstatement of nicotine place preference following U50,488 in the no stress group. Groups are defined in the figures as their final reinstatement grouping (U50 = U50,488). Data represent the mean preference (s) ± SEM, *n* = 5–8 (*p < 0.05, Student’s *t*-test). **(C)** Locomotor activity measured as distance traveled (cm) during three cocaine conditioning days. Data represent the mean preference (s) ± SEM, *n* = 5–8 (*p < 0.05, no stress vs. chronic mild stress on days 24 and 25, respectively; two-way ANOVA followed by Bonferroni *post hoc* test). **(D)** Locomotor activity measured as distance traveled (cm) during reinstatement. Data represent the mean preference (s) ± SEM, *n* = 5–8 (*p < 0.05 at 5 min and 10 min time point in the no stress group vs. CMS group; two-way ANOVA followed by Bonferroni *post hoc* test).

Kappa opioid receptor agonists and stress have been shown in numerous models to affect the magnitude of cocaine and nicotine place preference as well as cause reinstatement of cocaine and nicotine preference behavior (Redila and Chavkin, 2008; Land et al., 2009; Bruchas et al., 2010; Al-Hasani and Bruchas, 2011; Al-Hasani et al., 2013; Jackson et al., 2013). However, it is not known how repeated CMS ultimately influences cocaine and nicotine preference, or how CMS influences subsequent KOR or drug-primed reinstatement of cocaine or nicotine reinstatement. Therefore, we determined how a CMS paradigm adapted from Elizalde et al.(2008), Peng et al. (2012), and Tye et al. (2013) (**Table 1** and **Figures 3A** and **4A**) influences cocaine and nicotine conditioning, as well as KOR-induced reinstatement of cocaine and nicotine CPP. The CMS paradigm involved a random assignment of seven mild stressors during a 3-week period (see **Table 1**) prior to the cocaine or nicotine CPP/reinstatement training protocol.

There was no significant difference between the no stress and CMS-exposed groups in the magnitude of the initial cocaine (15 mg/kg, s.c.) or nicotine (0.5 mg/kg, s.c.) conditioned place preference scores. This finding suggests that exposure to CMS does not effect the subsequent conditioned rewarding effects of cocaine or nicotine (**Figures 3B** and **4B**). Extinction rates of place preference between no stress, SDS-exposed and CMS-exposed groups were not significantly changed. In contrast, following extinction of either cocaine or nicotine place preference in CMS groups, KOR-induced reinstatement of both cocaine and nicotine place preference was significantly reduced (**Figure 3B**: *p < 0.05, no stress and U50,488 vs. stress and U50,488; Student’s *t*-test, *n* = 7; **Figure 4B**: *p < 0.05, Student’s *t*-test, *n* = 5–8. While CMS protected against U50,488-induced reinstatement it had no significant effect on cocaine or nicotine drug-primed reinstatement; indicating that this protective effect of CMS was selective for KOR agonist-induced reinstatement. Locomotor activity was measured during the 3-day conditioning period to determine if exposure to 3 weeks of CMS alters the locomotor response to nicotine (0.05 mg/kg, s.c.) and cocaine (15 mg/kg, s.c.). No significant differences in locomotor activity were found between no stress group and the CMS group following cocaine treatment (**Figure 3C**). As predicted, distance traveled significantly increased over the 3-day cocaine conditioning period, as previously shown (Zhang et al., 2002; Tzschentke, 2007; **Figure 3C**: ^#^*p* < 0.05 day 23 vs. day 25 in the no stress group; **p < 0.01 day 23 vs. day 24 and ***p < 0.005 day 23 vs. day 25 between stress group; one-way ANOVA followed by Bonferroni *post hoc* test, *n* = 7). However, nicotine-induced elevations in locomotor activity were significantly reduced following CMS on the second and third day of nicotine conditioning (**Figure 4C**: *p < 0.05, no stress**vs. CMS as compared to the no-CMS group; two-way ANOVA followed by Bonferroni *post hoc* test, *n* = 5–8). Locomotor activity was also measured during U50-induced reinstatement in both the nicotine and cocaine groups. In the cocaine group only at the 5 min time point was there a significant decrease in locomotor activity in the CMS group compared to the no stress group (**Figure 3D**: *p < 0.05; two-way ANOVA followed by Bonferroni *post hoc* test). The nicotine group showed a similar profile, locomotor activity was also significantly decreased in the CMS group compared to the no stress group at both the 5 and 10 min time point (**Figure 4D**: *p < 0.05; two-way ANOVA followed by Bonferroni *post hoc* test). Together, these results support the conclusion that CMS protects against subsequent KOR-mediated cocaine and nicotine reinstatement and further suggests that CMS may act to regulate KOR system function.

## DISCUSSION

In the present study we investigated the interactions between various types of stress paradigms and how they influence KOR-induced reinstatement of cocaine and nicotine preference. We determined the effects of a single acute stress, sub-chronic social defeat, and CMS on cocaine and nicotine conditioned place preference and KOR-induced reinstatement. Although, FSS, SDS, and CMS have all been implicated in regulating hedonic state and reward, the present report describes a previously unknown connection between prior stress exposure and KOR-mediated stress-induced behavior. Together, these findings suggest that various types of stress exposure act to influence the dynorphin/KOR-mediated reinstatement response, but they also demonstrate that prior CMS has no lasting effect on the rewarding properties of cocaine or nicotine in conditioned place preference paradigms.

Stress-induced opioid peptide release has been reported for three major opioid systems, and its release has been demonstrated to be associated with a variety of behavioral outputs including stress-induced analgesia, reinstatement, dysphoria, depression, and anxiety-like behaviors (Carlezon et al., 2006; Land et al., 2008; Bruchas et al., 2010; Chartoff et al., 2012; Al-Hasani et al., 2013). In these reports, animals are typically exposed to a single stress event (e.g., forced swim, social defeat, foot shock) and then KOR-mediated behaviors are measured following acute stress exposure (McLaughlin et al., 2003; Bruchas et al., 2011; Schindler et al., 2012). However, the stress experience over the course of an animal’s lifetime is complex in nature occurring usually in multiple, randomized epochs. Therefore, the effects of CMS on KOR-mediated behavior remain an important area of investigation. Both repeated stress, and CMS models have been reported to induce dramatic changes in neural circuits and neuromodulator function (Hill et al., 2012; Lemos et al., 2012a, b; Tye et al., 2013), however, CMS effects on regulating cocaine-, nicotine-, and opioid-mediated conditioned preference behaviors is not known. Specifically, CMS-induced changes in serotonergic, noradrenergic, and dopaminergic signaling and release have all been reported (Rasheed et al., 2008; Vancassel et al., 2008; Hill et al., 2012) implicating important functions of monoamine regulation in CMS alterations of mood and reward seeking. In addition, it has been shown that repeated stress and CMS dramatically alter neuropeptidergic circuit function and impact neuropeptide modulation in subsequent stress responsivity, by either sensitizing the response or acting to facilitate desensitization. In the current report we found that following a single acute FSS, activation of KOR signaling caused potentiation of cocaine preference-reinstatement (**Figure 1**). In contrast, repeated SDS or CMS protected against subsequent KOR-mediated reinstatement (**Figures 2, 3**, and **4**). We also found that this CMS protective effect not only influenced KOR-induced cocaine reinstatement (**Figure 3**) but it also blocked KOR-induced reinstatement of nicotine place preference (**Figure 4**), suggesting a conserved mechanism for multiple drugs of abuse. Interestingly, CMS exposure had no effect on the initial cocaine or nicotine conditioning, nor did CMS exposure impact the magnitude of drug-primed reinstatement. These findings suggest that SDS and CMS induce a type of “tolerance” to subsequent KOR activity, which has also been previously reported when administering repeated doses of KOR agonists (McLaughlin et al., 2004). Stress adaptability mechanisms are a critical consideration in our current findings because reports demonstrating that dramatic changes in neural circuit function include effects on KOR and corticotropin releasing factor (CRF) signaling following various types of repeated stress exposure (McEwen and Gianaros, 2011; Lemos et al., 2012a, b).

The precise neuronal and molecular mechanisms for SDS- and CMS-induced attenuation of KOR-induced reinstatement were not identified in this study but are important and exciting extensions of this work. There are a number of mechanisms that maybe involved in CMS-induced regulation of KOR-reinstatement. KOR regulation following repeated and high agonist-receptor occupancy has been previously reported to be mediated by GRK3 phosphorylation of serine 369 in mouse/rat KOR or serine 358 in the human KOR (Wang et al., 2003; McLaughlin et al., 2004; Bruchas et al., 2006, 2007a; Chen et al., 2007). In repeated SDS or CMS models it is possible that recurrent activation of KOR causes subsequent downregulation and desensitization of KOR signaling preventing further activation of KOR until full recovery. Furthermore, KOR regulation and deactivation (in contrast to mu-opioid receptors) take several weeks to recover as functional receptor entities (McLaughlin et al., 2004; Bruchas et al., 2007b), consistent with the CMS time course used in this study. Surprisingly the preventative effect of prior stress on KOR-mediated reinstatement did not also prevent KOR-induced decreases in locomotor activity (**Figure 2C**) suggesting that SDS acts in specific neural circuits associated with reinstatement but not locomotion. Therefore selective blockade of KORs in specific brain regions during repeated SDS, and CMS and subsequent measures of KOR-mediated behavioral responses will be required in future studies. In addition, utilizing GRK3 knockout mice, or expression of KOR-S369/358 mutants *in vivo*, and exposure to CMS are interesting extensions of this work as prior work has shown that GRK3 signaling is required for KOR-mediated behavioral effects via phosphorylation, arrestin recruitment, and p38 signaling (Bruchas et al., 2011). It has also been shown that KOR-mediated signaling to G-protein inwardly rectifying potassium channels in the serotonergic dorsal raphe nucleus (DRN) is altered following repeated stress exposure (Lemos et al., 2012a). Together, with the recent evidence that these circuits are implicated in KOR-induced reinstatement (Land et al., 2009; Bruchas et al., 2011) it is possible that CMS causes dysregulation of KOR signaling in the DRN to ultimately influence subsequent KOR-induced behavioral responses including reinstatement of drug seeking. Furthermore, CMS may cause downregulation of KOR in the locus coeruleus, a region which has recently been identified to play a key role in KOR-mediated reinstatement to cocaine place preference (Al-Hasani et al., 2013). However, these hypotheses will require further study using selective neural circuit dissection techniques.

The effects of stress on cocaine and nicotine seeking have been widely reported by numerous groups (for reviews see Koob, 2008; Aguilar et al., 2009). In the case of cocaine, it is well-established that stress and exposure to cues causes robust reinstatement of drug seeking in both animals models and human subjects (Koob and Kreek, 2007). The effects of stress on nicotine reinstatement in animal models are less well-characterized, although reports have shown that acute stress exposure potentiates nicotine-seeking behavior and reinstates drug seeking (Buczek et al., 1999; Bilkei-Gorzo et al., 2008; Plaza-Zabala et al., 2010; Yamada and Bruijnzeel, 2011; Leão et al., 2012; Smith et al., 2012). It has also been established that human subjects widely report stress as the primary reason for their continued tobacco use (Bruijnzeel, 2012). However, the effects of randomized CMS on nicotine place preference in animal models has not been previously studied, nor have mechanisms of stress on KOR-induced regulation of nicotine-induced behavior been widely explored with the exception of some recent reports implicating KOR in nicotine-induced behavior (Smith et al., 2012; Jackson et al., 2013). Our current findings build on this prior work, and for the first time show that agonist-induced KOR activation is sufficient to cause reinstatement of nicotine CPP. Additionally, our finding that KOR agonist does not promote reinstatement of nicotine CPP following CMS suggests interesting and complex interactions between CMS and dynorphin/KOR neural circuits that are conserved for both nicotine and cocaine. However, whether similar neural circuits (e.g., dopaminergic, serotonergic) are required for KOR-dependent nicotine reinstatement as compared to cocaine reinstatement will require further investigation.

 In summary, we report that frequency and duration of stress differentially influences KOR-induced reinstatement of cocaine and nicotine preference. The present study shows that repeated stress or CMS prior to reinstatement prevents a KOR-induced reinstatement response, while acute exposure to stress induces potentiation of KOR-reinstatement. The wide array of recent studies investigating the interactions between stress and KOR function on reward, reinstatement, and dysphoria suggest that KOR interacts with multiple neurotransmitter systems and circuits to mediate its complex role in behavioral output. We identified previously unrecognized roles for acute, sub-chronic, and chronic stress exposure on KOR-mediated behavioral function. These findings suggest that understanding the regulation of dynorphin/KOR systems in response to various stress exposures is critical to understanding and identifying KOR as a potentially novel therapeutic target system in drug relapse, anxiety, and depression.

## Conflict of Interest Statement

The authors declare that the research was conducted in the absence of any commercial or financial relationships that could be construed as a potential conflict of interest.
